# An investigation into the influence of context effects on crowd exit selection under gender difference in indoor evacuation

**DOI:** 10.3389/fpsyg.2024.1417738

**Published:** 2024-07-10

**Authors:** Qi Teng, Xuan Wang, Wu He, Gaofeng Pan, Yan Mao

**Affiliations:** ^1^School of Business, Sichuan Normal University, Chengdu, China; ^2^School of Psychology, South China Normal University, Guangzhou, China; ^3^College of Movie and Media, Sichuan Normal University, Chengdu, China; ^4^The School of Cyberspace Science and Technology, Beijing Institute of Technology, Beijing, China

**Keywords:** exit choice, emergency evacuation, context effect, gender, crowd behavior

## Abstract

**Introduction:**

Exit selection is crucial in indoor emergency evacuation. Domestic and foreign scholars have found that exit choice behavior is influenced by three factors: environmental factors, social interactions, and individual internal factors. Previous studies have shown that in addition to a single environmental factor affecting exit decisions, the influence of other available exit options in the context can ultimately lead to a reversal of exit decisions -The context effect. However, the impact of context effects on exit decisions in emergency situations has not been thoroughly explored. Therefore, this article identifies three basic independent variables: context effects, crowd flows, and gender differences, to study the exit decisions of different gender groups facing different crowd flows, as well as how context effects affect existing exit decisions.

**Methods:**

In this paper, we used virtual reality technology to construct an indoor fire scene and designed a total of 15 virtual experiments with different crowd distribution or context effects. 131 participants were divided into two groups, male and female, and their exit decisions were observed under different crowd flows and contextual effects.

**Results:**

The research results show that: 1) Both men and women have an innate preference to avoid crowded exits, and the proportion of following crowd evacuation significantly decreases when there are crowded crowds in the scene; 2) The exit decisions of female participants are more influenced by the crowd, while men tend to be more influenced by context effects when evacuating independently; 3) The context effects on exit decisions in emergency situations is statistically significant, and this performance is more significant in the male population. Further analysis reveals that similarity effects have a more significant impact on exit decisions than attraction effects.

**Discussions:**

These findings provide deeper insights into the exit choice behavior of the population and may contribute to the design of safe exits in indoor buildings. In addition, this article emphasizes the importance of context effects and provides a foundation for future research.

## 1 Introduction

Fire has always been a typical indoor emergency situation, often threatening people’s lives and safety. According to data released by the National Fire and Rescue Bureau, a total of 550,000 reported fires were reported nationwide in 2023, with 1,311 people injured and 959 deaths, resulting in direct property damage of 3.94 billion yuan. Timely and rapid evacuation in the face of indoor fires can effectively reduce casualties and rescue losses, so exit selection is the key in evacuation (Song and Lovreglio, 2021).

When conducting emergency evacuation in indoor buildings, pedestrians often have to face multiple exits and choose which one to ultimately use for evacuation (Heliövaara et al., 2012), understanding this pedestrian exit selection behavior is crucial for emergency evacuation research (Kobes et al., 2010). Domestic and foreign scholars have found that pedestrian exit selection is a complex decision-making process (Farr et al., 2012), which is mainly influenced by three aspects: social factors, environmental factors, and personal factors (Zhu et al., 2020). Specifically, social factors refer to the leader following behavior generated by pedestrians following authoritative figures during evacuation (Haghani et al., 2020), or the herd effect exhibited during crowd evacuation (Haghani and Sarvi, 2016, 2019a,b); Environmental factors are mainly manifested in the positive or negative effects of indicator signs (Vilar et al., 2013; Feng et al., 2021; Zhao et al., 2022), exit settings (Kurdi et al., 2018; Tong and Bode, 2023), exit visibility (Kobes et al., 2010; Wang et al., 2022), and fire hazards (Lovreglio et al., 2016; Song and Lovreglio, 2021) on crowd evacuation in buildings; Personal factors mainly include the familiarity of participants with exits (Kinateder et al., 2018; Jia et al., 2020), personal risk preferences (Xue et al., 2010; Fu et al., 2021), and gender (Huang et al., 2022), which have different impacts on exit decisions. However, early research reports on evacuation suggested that people may experience panic in the event of a fire, resulting in the crowd not always integrating all factors within the scene to make the most rational exit decision (Klüpfel et al., 2005; Duives and Mahmassani, 2012). People’s exit choices exhibit certain preferences and may have strong subjective preferences for a certain factor due to individual differences (Gao et al., 2022). This exit choice preference still needs further exploration in the field of evacuation.

in social factors, the performance of the herd effect in emergency evacuation has been widely studied by scholars. The herd effect refers to a collective behavior exhibited during crowd evacuation (Raafat et al., 2009), which reflects a preference to follow the crowd in making exit choices. Previous studies have found that social attributes of the population (Hu et al., 2012; Zhu et al., 2020; Haghani, 2020; Zhu et al., 2020), unfamiliarity with evacuation spaces (Haghani and Sarvi, 2017), visual limitations (Meng et al., 2019), and stress emotions during emergency evacuation (Alrashed and Shamma, 2020) may all lead to herd behavior becoming a preferred factor for people’s exit choices. The performance of herd behavior in the field of emergency fire evacuation can be mainly divided into following the majority of people to make exit choices (Haghani and Sarvi, 2019a,b) or avoiding following the crowd to make exit decisions (Haghani and Sarvi, 2019a,b). People’s exit choice changes depend on the distribution of the crowd (Tong and Bode, 2023). Research has found that the performance of the herd effect in emergency evacuation is also different. Some scholars believe that the crowd congestion caused by the herd effect in emergency evacuation hinders evacuation efficiency (Haghani and Sarvi, 2019a,b), but other studies have found that the generation of this effect can reduce people’s reaction time (Haghani and Sarvi, 2019a,b) and improve evacuation efficiency (Vecliuc et al., 2022), especially in situations where vision is obstructed (Meng et al., 2019) or when a fire generates a lot of stress emotions (Alrashed and Shamma, 2020) In the state of. This reflects the significant background specificity of the herd effect, which can have different consequences under the influence of evacuation situations. Therefore, it is important for this article to consider the impact of the herd effect on exit choices from the perspective of gender differences between men and women.

Meanwhile, studies have found that context can promote or hinder the evaluation of environmental factors, thereby shaping the preferences of decision-makers (Tong and Bode, 2023). This preference leads them to make different exit choices under the influence of context factors, which is the context effect (Gao et al., 2023). Generally speaking, preference changes caused by context effects, that is, the choice of preferred exits depends on the availability of alternative exits (Trueblood et al., 2012; Cataldo and Cohen, 2019), are reflected in three main effects: compromise effect (Itamar, 1989), similarity effect (Tversky, 1972), and attraction effect (Klüpfel et al., 2005). In the compromise effect, when the same exit option is seen as a compromise between other options, it is considered more attractive than when it is an extreme option (Trueblood et al., 2014). Similarity effect refers to adding a similar exit (not significantly lower or superior) to an existing exit option, where participants make exit choices from similar options and are guided to different options (Spektor et al., 2019). The attraction effect refers to the increase in the probability of selecting the better option when an option similar to but lower than another option is added to the selection set (Trueblood et al., 2012). The influence of context effects has been widely applied in consumer purchasing (Doyle et al., 1999), choice problems (Bateson and Healy, 2005), and perception problems (Trueblood and Pettibone, 2017). In the field of emergency evacuation, researchers have explored the impact of three main effects on participant exit choices (Gao et al., 2023). However, this study has not delved into the performance of context effects in the field of emergency evacuation, and has not yet explained how context effects interact with other factors and how they affect crowd exit decisions. Therefore, this article will distinguish the impact of context effects on exit choices of different gender groups when studying context effects, and consider the factor of herd effect to further understand how context effects affect people’s exit decision-making preferences in the field of emergency evacuation.

Finally, gender as a individual factor is an important internal factor that influences evacuation behavior and has a significant impact on people’s exit decisions. Psychological research has found that men are better at regulating emotions (Chaplin, 2015), while women have an advantage in emotion recognition and a stronger susceptibility to negative emotions. Therefore, during emergency evacuation, men are more calm (Pan et al., 2021) and women are more likely to exhibit panic emotions (Lu et al., 2011), and this different emotional processing method also leads to different evacuation behaviors (Lu et al., 2011). Domestic and foreign researchers have found that gender effects have a significant impact on people’s average evacuation speed (Huang et al., 2022), spatial perception style (Xiaohui et al., 2019), information search ability (Shiwakoti et al., 2016), and following behavior (Feng et al., 2020) during emergency evacuation. In the process of exit selection, men are more adventurous than women (Lipps et al., 2013) and have a stronger ability to accept environmental information (Shiwakoti et al., 2016). Therefore, when the herd effect occurs, men are more willing to avoid the crowd (Feng et al., 2020) and choose exits based on indicator signs (Lu et al., 2011), while women show a stronger willingness to follow the crowd and make exit decision preferences (Zhu et al., 2023). This article will investigate how the herd effect affects exit decisions from different gender perspectives, and whether context effects alter exit decisions of different genders.

Virtual reality (VR) technology is increasingly being used to study exit/route selection behavior in evacuation (Haghani et al., 2020). Through virtual reality experiments, the safety of the behavior of research participants in emergency situations can be ensured (Duarte et al., 2014), while improving the control of the experiment (Kinateder et al., 2014) to more accurately analyze the impact of different factors on pedestrian behavior (Max and Warren, 2016). The ecological effectiveness of this technology has also been demonstrated in the experiment (Max and Warren, 2016). Environmental factors are a focus of research by domestic and foreign scholars using VR technology in the field of emergency evacuation. They have extensively explored the effects of exit signs (Vilar et al., 2014; Zhang et al., 2021), fire conditions (Tucker et al., 2018; Cao et al., 2019), visual factors (Zhu et al., 2020), and other factors on exit selection. Another research focus of VR technology in the field of emergency evacuation is the social interaction effects among crowds (Lin et al., 2020). By simulating a large number of crowds through VR, the impact of crowd effects on emergency evacuation can be studied in a safer way (Li et al., 2019). So far, a large number of studies have confirmed the availability and effectiveness of virtual reality methods in the field of emergency evacuation (Bourhim and Cherkaoui, 2020). This article will also use virtual reality technology to construct emergency evacuation scenarios to study how context effects affect the exit decision-making behavior of different gender groups in the face of crowd effects.

In summary, this article aims to use virtual reality technology to specifically study how the exit choices of different gender groups are influenced by the herd effect in emergency fire evacuation situations, whether this choice will be further influenced by context effects, and how context effects interact with herd effects to affect the exit decisions of the population. Through research, we can further investigate the impact of situational effects on people’s decision-making in fire evacuation, as well as how and to what extent different gender groups are affected. By recording people’s exit decisions and evacuation efficiency, this article effectively explores the impact of herd effect and situational effect on people’s decision-making and behavior under the interaction of gender factors. Among them, we specifically explored the impact and intensity of similarity effects and attraction effects on decision-making through situational effects. Detailed explanations will be provided in the following chapters.

## 2 Method

### 2.1 Experimental design

This study adopted an inter-group experimental design and controlled for three independent variables: crowd flows (the crowd flow: 25 NPC/the uncrowded flow: 10 NPC), gender factor (exit decision-making of different gender groups), and context factor (the influence of similarity and attraction effects on exit decision-making).

#### 2.1.1 Crowd flows

The independent variable of crowd flows is reflected in the distribution of pedestrians near the exit, so as to understand whether participants are affected by the herd effect. In a crowded state of crowd distribution, the simulated crowd will gather at the exit to form a crowded scene, and participants may form a herd mentality and choose to follow the crowd to the exit; Or avoid crowding and reduce the use of the exit. In the uncrowded state, the crowd is freely distributed near the exit, and participants may develop a risk averse mentality, avoiding the unselected exit and choosing to follow the crowd to the exit. Before the formal experiment began, we reviewed the previous studies and conducted a preliminary experiment to statistically investigate the participants’ perception of the population size. Based on the data from the pilot experiment, more than 90% of people believe that a population of less than 10 people is considered non congested, while a population of 25 people is considered congested. We ultimately determined that the number of participants who considered the crowd to be crowded was 25, and the number of non-crowded people was 10. This data shows in the Table 1 and will be used for subsequent experimental design.

**Table 1:** Preliminary experiment to statistic the participants’ perception of the crowd size.

The crowd flows	Number of selections	Percentage
Under 10 peoples	7	5.34%
11–15 peoples	40	30.53%
16–20 peoples	44	33.59%
21–25 peoples	22	16.79%

**Figure 1:**
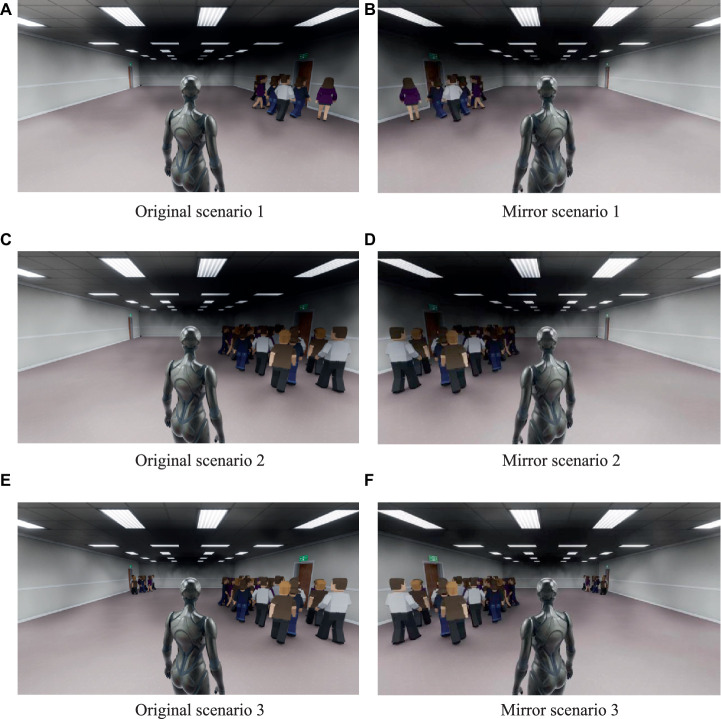
The virtual experiment scenarios under different crowd flows in experiment 1: Scenario 1 means the crowd flow is 0 vs. 10; Scenario 2 means the crowd flow is 0 vs. 25; Scenario 3 means the crowd flow is 10 vs. 25. The figure **(A, C, E)** shows the original scenarios. The figure **(B, D, F)** shows the mirror scenarios, which is symmetry with the exit settings of the original scenarios.

Therefore, the crowd movement patterns are divided into three scenarios, namely (1) 0 vs. 10, with no NPC distributed at exit A that is farther away, and 10 NPCs distributed at exit B that is closer, which is a non-crowded situation. (2) 0 vs. 25, with no NPC distributed at exit A that is farther away, and 25 NPCs distributed at exit B that is closer. At this point, exit B is defined as a congested exit. (3) 10 vs. 25, 10 NPCs are distributed at exit A, which is farther away, and 25 NPCs are distributed at exit B, which is closer. At this time, the crowd at both exits is unevenly distributed, and exit B is still crowded. The precise movement mode used to simulate the number of rows varies according to the experimental scenario, as shown in Figure 1. Unity’s physics engine controls the movement of simulated crowds, allowing them to move, react to collisions, and form a distribution in a physical reality manner. The population flow variable is mainly used to study the impact of herd effect on participants’ exit choices.

#### 2.1.2 Gender factors

Gender factors are mainly reflected in the final impact of different genders of participants on exit decision-making. For example, during emergency evacuation, due to gender differences, female participants can choose more exits that follow the distribution of the crowd for emergency evacuation. Therefore, we take into account the gender of participants, a variable primarily aimed at investigating the impact of gender differences in group effects on participant exit choices.

#### 2.1.3 Context effect

The generation of context effects is closely related to the existence of alternative exit sets within the scene. The context effect is mainly reflected in the impact of adding an alternative exit in the scene on the exit selection of participants in emergency evacuation situations. For example, under the influence of similarity effects, exits are similar to original exits, which weakens participants’ preferences for existing exports and leads to diversion of exit choices; Under the attraction effect, the addition of new exits is not as good as the original exits, but instead strengthens the preferences of participants toward the original exports. Therefore, we divide context effects into two different scenario designs: (1) Under similar effects, there are exits in the scenario that are similar to the original exit, in order to investigate whether it weakens participants’ selection of the original exit (C_A_ represents new exit C similar to original exit A, C_B_ represents new exit C similar to original exit B). (2) Under the attraction effect, there are inferior exits in the scene compared to the original exits, in order to investigate whether it will strengthen participants’ selection of the original exits (C_A_ represents the inferior exits of the new exit C to the original exit A, C_B_ represents the inferior exits of the new exit C and the original exit B).

#### 2.1.4 Similarity effect

**Figure 2:**
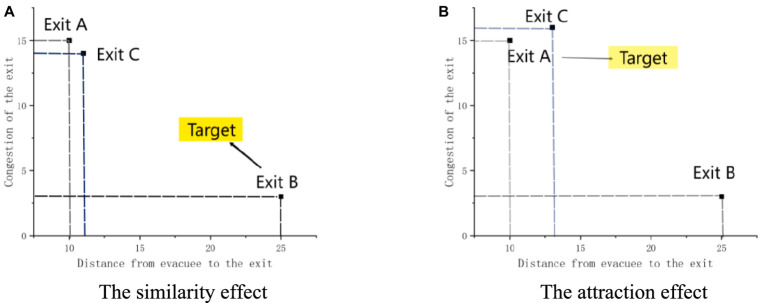
The context effect. **(A)** Shows the similarity effect. **(B)** Shows the attraction effect.

Add a new exit C in the scene that is similar to the original exit. Compared to the original exit, the new exit has similar characteristics and is a good alternative to use. Participants will be affected by the addition of similar exits, and some may choose to use the new exit C. For example, as shown in Figure 2A, If exit A is similar to new exit C, then they exhibit similar exit characteristics. In this study, it refers to a similar flows of crowd and exit distance. When Exit C exists, it will lead to the diversion of people who originally chose Exit A, while Exit B is not affected by similar effects and becomes the target exit for more people to choose.

#### 2.1.5 Attraction effect

Add a relatively low-quality exit C in the scene, which is not as good as the original exit in terms of characteristic performance. Participants are affected by the new exit and compared with the original exit, which actually strengthens their choice of the original exit. Fewer participants consider the new exit C as an alternative choice. For example, as shown in Figure 2B, Exit C is slightly larger in distance and congestion than Exit A. In the scene, Exit A is considered a low-quality exit. At this point, the presence of Exit C will guide the crowd to have lower crowding levels and shorter distances compared to Exit A. Exit A becomes a more evacuation exit, leading to Exit A becoming the target exit.

#### 2.1.6 Experimental design

**Figure 3:**
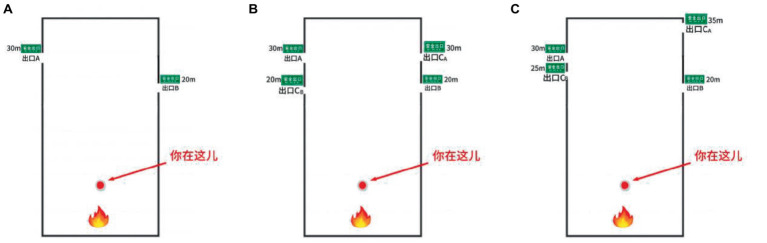
The scene plan: **(A)** the basal virtual scene plan; **(B)** the similarity effect scene plan; **(C)** the attraction effect scene plan.

**Figure 4:**
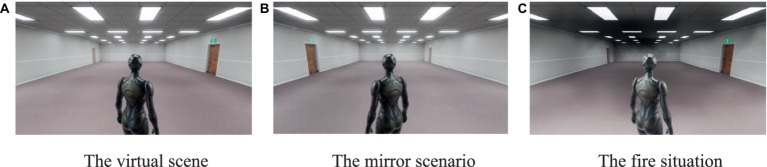
The virtual scenarios. **(A)** shows the exits settings under basic virtual scenarios. **(B)** shows the exits settings under the mirror scenario. **(C)** shows the fire situation.

Experiment 1 and Experiment 2 follow the same subjects and experimental procedures. There are differences in the experimental scenarios. Experiment 2 has added a new exit on the basis of the original scenario to reflect the impact of different contextual effects (the experimental scenario floor plan is shown in Figure 3, and the virtual scenario is shown in Figure 4).

##### 2.1.6.1 Experiment 1

The virtual testing environment is a rectangular indoor building with two exits, and there are safety evacuation signs in front of it. The distance from the starting point to the farthest Exit A on the left is 30 m, and to the nearest Exit B on the right is 20 m. The two exits are not equal in distance to the participants and have different crowd flows (as shown in Figure 1). At the same time, in order to reduce the influence of participants’ left and right habitual preferences on exit selection decisions, we set up a mirror experiment scenario to avoid this error. The mirror experiment scenario is no different from the normal experiment scenario except for symmetrical left and right exits. Therefore, in Experiment 1, participants were required to complete a total of 6 different experimental scenarios as shown in Table 2. This experiment investigated whether crowd behavior (crowd flows) and gender factors would affect the exit choices of participants during emergency evacuation.

**Table 2:** The experiment 1 scenarios.

Variables		Original scenarios		Mirror scenarios	
Gender difference	Crowd split	Left exit A	Right exit B	Left exit A	Right exit B
Male	0–1 split	0	10	10	0
	Uneven	0	25	25	0
		10	25	25	10
Female	0–1 split	0	10	10	0
	Uneven	0	25	25	0
		10	25	25	10

*H0*: Crowd behavior and gender factors do not affect participants’ exit choice.

*H1*: Crowd behavior and gender factors affect participants’ exit choice.

##### 2.1.6.2 Experiment 2

**Figure 5:**
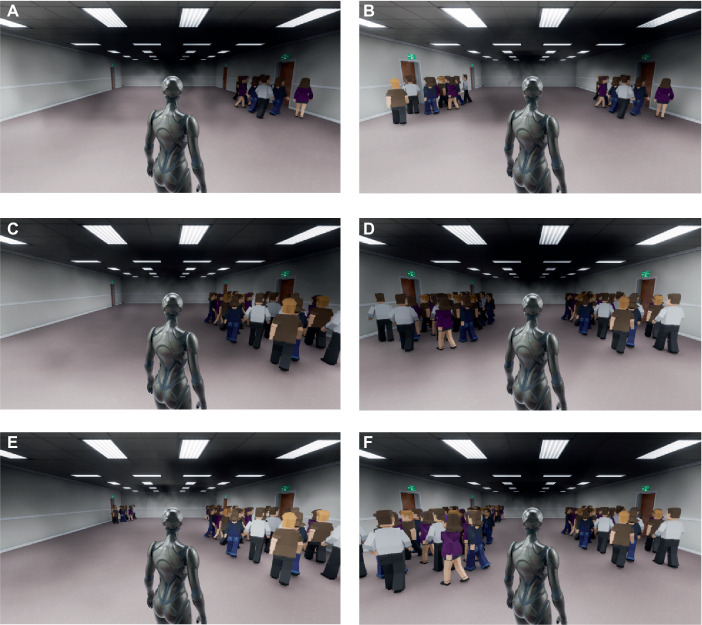
The similarity effect shows in the virtual scenarios: **(A,C,E)** shows there is an exit C_A_ similar to the exit A; **(B,D,F)** shows there is an exit C_B_ similar to the exit B.

**Figure 6:**
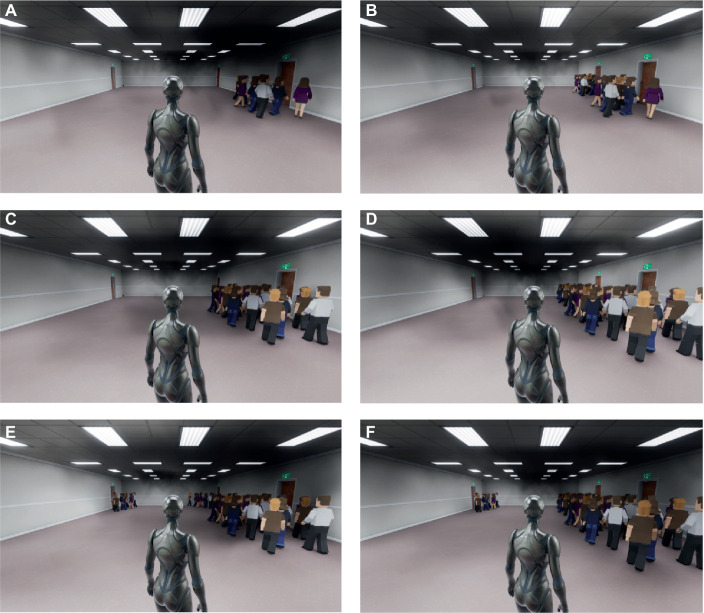
The attraction effect shows in the virtual scenarios: **(A,C,E)** shows there is an exit C_A_ inferior to the exit A; **(B,D,F)** shows there is an exit C_B_ inferior to the exit B.

The basic building setup is the same as Experiment 1, but a new exit C has been added based on the performance of context effects. When Exit C_A_ is similar to Exit A, Exit C_A_ has the same crow flow as Exit A, with a distance of 30 m from the starting point; When Exit C_B_ is similar to Exit B, there is the same crowd flow between Exit C_B_ and Exit B, with a distance of 20 m from the starting point; When Exit C_A_ is a low-quality exit of Exit A, there are more people distributed near Exit C_A_ than near Exit A, with a distance of 35 m from the starting point; When Exit C_B_ is inferior to Exit B, there are more people distributed near Exit C_B_ compared to Exit B, with a distance of 25 m from the starting point. Participants are required to complete 12 experiments in different scenarios, and the specific experimental design is shown in Table 3. This experiment investigated whether context effects, crowd flow, and gender factors would affect the exit choices of participants during emergency evacuation (Figures 5, 6).

**Table 3:** The experiment 2 design.

Variables	Description of new exit C	Crowd distribution		
		Exit A	Exit B	Exit C
The similarity effect	The exit C is similar to A	0	10	0
		0	25	0
		10	25	10
	The exit C is similar to B	0	10	10
		0	25	25
		10	25	25
The attraction effect	The exit C is inferior to A	0	10	0^+^
		0	25	0^+^
		10	25	10^+^
	The exit C is inferior to B	0	10	10^+^
		0	10	10^+^
		10	25	25^+^

*H0*: Context effects, crowd flows and gender factors do not affect participants’ exit choices.

*H1*: Context effects, crowd flows and gender factors influence participants’ exit choices.

### 2.2 Participants

**Figure 7:**
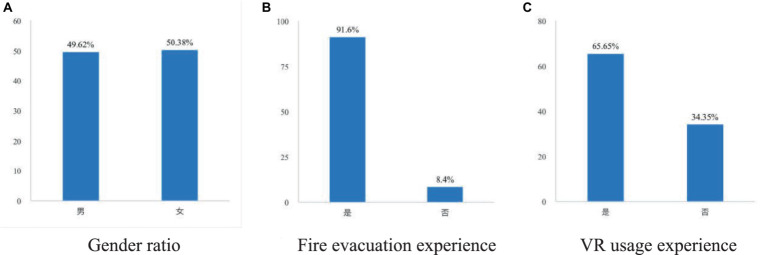
Participant information statistics.

A total of 131 participants were recruited for Experiment 1 and Experiment 2 of this study. Among them, there are 65 males and 66 females. They are all normal or corrected to normal, with normal color vision. Through investigation, we asked participants about their gender, fire evacuation experience, and VR device usage experience, as shown in the Figure 7. Each participant received 20 RMB.

The Ethics Committee of Sichuan Normal University approved the research plan in accordance with the Helsinki International Declaration. The study participants received verbal consent, and participation was entirely based on their wishes. The participants were also informed of the purpose of the study and its procedures. In addition, participants were informed that they had the right to withdraw from the study.

In the process of collecting experimental data, males and females were classified by gender, based on the gender of the basic sexual information filled in the demographic scale.

### 2.3 Virtual environment

This experiment uses virtual reality technology to study the exit selection, evacuation speed, and time of pedestrians in different situations. Participants are placed in a first person view of a 3D virtual environment and can control the movement of a simulated human in the environment by using a joystick to move forward, backward, left, and right. Non player characters are pedestrians manipulated by participants in virtual reality experiments. This means that non player characters are the main body of the game itself and are only replaced by non-player characters in the virtual reality world. Non player character movement is when participants control the movement of non-player characters (themselves) in the virtual world through a joystick. For example, if the subject manipulates the joystick to move left, non-player characters in the virtual world will exhibit the same movement behavior (i.e., moving left). The presence of virtual pedestrians allows participants to determine their position relative to other pedestrians in the room. This experiment includes a training phase and an exit selection phase. During the training phase, participants are allowed to move freely in the room to become familiar with controlling the movements of non-player characters. In the exit selection stage, participants must complete a simulated fire emergency evacuation under experimental conditions.

This study was conducted in a virtual reality laboratory. We used the HTC VIVE Head Mounted Display (HMD) VR system with Steam VR positioning technology, which allows for a 360^o^ view display. The field of view is 110^o^, and the resolution of the display is 1,080 (horizontal) x 1200 (vertical) pixels. This experiment was run on a Dell Precision T7800 workstation, equipped with an Intel Xeon E5-2603 processor and GBGTX1080 graphics card. We use 3D Studio Max software to model and render Ive (Immersive Virtual Environment), and then import it into the platform of Unity3D game engine. Using Unity3D to build a virtual environment, participants control virtual characters and non-player characters using the same Unity character package for animation production. This is mainly to reduce the possible impact of virtual characters on participants’ exit choices. The walking and rotating speeds of simulated pedestrians in the simulated world are set to 1.5 m/s and 80 degrees/s, respectively.

During the training phase, participants received instructions to use joysticks to control non player characters and were able to move freely in a rectangular room. The safety exit sign will be directly displayed above the exit in the room, so that participants can have a clearer understanding of controlling the direction and speed of non-player characters. Only when participants are confident that they can control the movement of virtual characters will they enter the exit selection stage. The actions of participants in the virtual environment during the training phase were not recorded.

In the exit selection stage, the simulator controlled by the participants is in a room with two exits. The distance between non player characters and the two exits is not equal, and the safety sign above the exit is displayed in high brightness to ensure that participants can fully see the exit and the area in front of the exit before starting to move. Participants were exposed to fire and smoke and received the following instructions: “Sudden fire. Please choose an exit to leave as soon as possible.” At this point, participants will see simulated pedestrians of different distributions evacuate through the exit and choose between the two exits. Once the participants reach the outside of one of the exits, the exit selection stage of the experiment ends. At this stage, we recorded the exit selection, reaction time, and evacuation time of participants in the virtual environment.

### 2.4 Procedure

**Figure 8:**
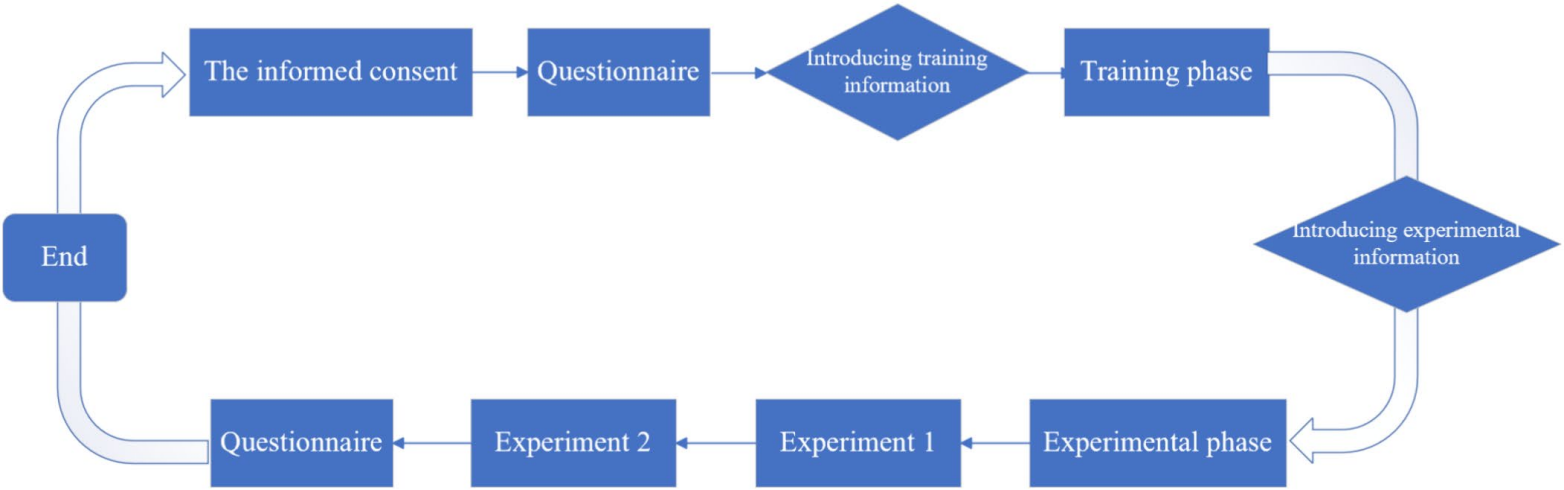
Procedure.

A total of 131 participants were recruited for this experiment, and the experimental process is as follows (Figure 8):

Firstly, the participants arrived at the laboratory and signed an informed consent form. Subsequently, participants were asked to complete a pre experiment questionnaire, which included basic demographic information, perception of crowd density in crowd flows, Positive and Negative Attitudes Scale (PANAS), and Virtual Reality Sick Questionnaire (VRSQ).

Then, participants were asked to complete training tasks and an exit selection before the experiment. The training task means that participants need to familiarize themselves with the environment, operate the equipment, and then have a 15 min break for formal experiments (exit selection).

After that, in the formal experiment, participants were required to complete emergency evacuation tasks that included different crowd flows and exit settings. There is a fire and smoke between the participants and the exit, and participants are required to make exit choices when facing the fire and smoke.

At the end of the experiment, each participant answered a post test questionnaire. The survey questionnaire includes feedback and ideas on virtual reality systems. It includes ANAS and VRSQ questionnaires.

## 3 Results

### 3.1 Data collection and analysis

A total of 131 data points were collected within the specified maximum time. The gender distribution of participants includes 66 females (50.38%) and 65 males (49.62%). Evaluate participant behavior by collecting the time required to reach the exit, their speed, and the type of exit chosen.

### 3.2 Exit choice

Multivariate logistic regression analysis was conducted on three independent variables and their interactions to investigate their impact on participants’ exit choices. The results are shown in Table 4. The results indicate gender effects [χ^2^(2) = 9.624, Sig = 0.010], context effect [χ^2^(8) = 123.531, Sig = 0.000], crowd flows [χ^2^(4) = 225.906, Sig = 0.000] has a significant impact on the exit choices of participants, therefore it is assumed that H1 is valid and H0 is rejected. These three effects have independent effects on the exit choices of participants, and together they can be used to predict the exit choices of 82.6% of participants in this study.

**Table 4:** Multivariate logistic regression regarding the participants’ exit choice.

Predictor	χ^2^	df	Sig
Intercept	0.000	0	NA
Gender	9.264	2	0.010
The context effect	123.531	8	0.000
Scenarios difference-Crowd flow	225.906	4	0.000
Goodness-of fit test	53.069	44	0.164
Correct percent of prediction	82.6%		

Goodness-of-fit test was based on deviance. β, SE β and e^β^ were different for regression model of each route and hence were not reported here in detail. Cox and Snell R2 was 0.166.

Finally, we conducted a study on whether participants had innate preferences for choosing left or right exits, and Table 5 shows the difference in exit selection between the normal scene and the mirror scene. It was found that in scenario 1, *t* = −0.556 and Sig = 0.579, in scenario 2, *t* = 0.000 and Sig = 1.00, and in scenario 3, *t* = 0.332 and Sig = 0.740. There was no significant difference in exit decisions between participants in the mirror scene and the normal scene (Sig > 0.05), indicating that participants did not exhibit innate left–right preferences. Therefore, we merged all the data in the following analysis and did not explicitly state that the left or right exit has higher utility or is used by more simulated pedestrians.

**Table 5:** Paired tests of mirror scenarios versus ordinary scenarios.

Scenarios	σ	S.E.	95%CI		*t*	Sig
			Lower	Upper		
Scenario 1	0.472	0.041	−0.104	0.059	−0.556	0.579
Scenario 2	0.215	0.019	−0.037	0.037	0.000	1.000
Scenario 3	0.263	0.023	−0.038	0.053	0.332	0.740

Scenario 1 represents there is no people near the Exit A, 10 people near the Exit B. Scenario 2 represents there is no people near the Exit A, 25 people near the Exit B. Scenario 3 represents there is 10 people near the Exit A, 25 people near the Exit B.

### 3.3 Gender effect

**Figure 9:**
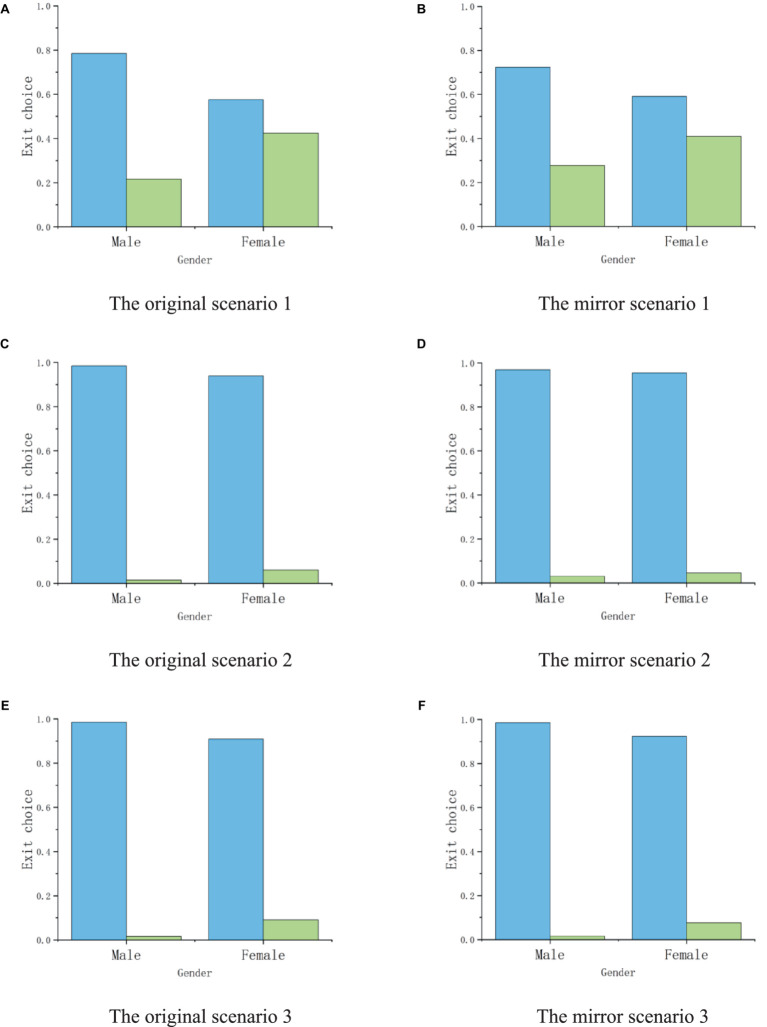
The exit choice proportions of experiment 1. **(A,C,E)** shows the exit choice under the original scenarios. **(B,D,F)** shows the exit choice under the mirror scenarios.

**Figure 10:**
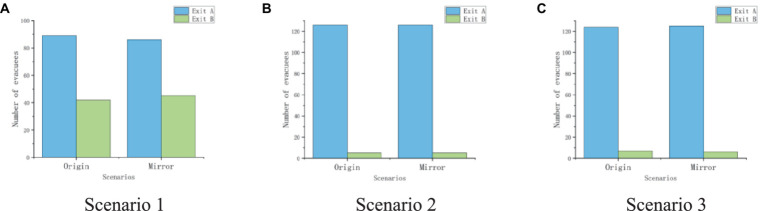
The statistics of exit choice in original scenarios and mirror scenarios. **(A)** shows the exit choice in scenario 1; **(B)** shows the exit choice in scenario 2; **(C)** shows the exit choice in scenario 3.

Figure 9 specifically shows the impact of gender effects on exit selection in six scenarios of Experiment 1. In scenario one without crowded crowds, 42.42% of women chose to follow the crowd to evacuate, while only 21.54% of men made the same decision. Even in situations where there is a crowded crowd in the scene, it can be observed that (*P*_2 male_ = 1.54% < *P*_2 female_ = 6.06%; *P*_3 male_ = 1.54% < *P*_3 female_ = 9.09%), women show a stronger willingness to follow the crowd for exit choices than men, while men are more willing to avoid the crowd and make exit choices (Figure 10).

Secondly, the impact of gender effect on population effect was analyzed through one-way ANOVA, and the analysis results are shown in Table 6. In scenario 1, where there are no people distributed at Exit A on the left and 10 people distributed near Exit B on the right, *F* = 6.799 and *p* = 0.01, gender factors have a significant impact on exit selection; Scenario 2 results showed that *F* = 1.822, *p* = 0.179, and participants of both genders avoided crowded Exit B more and chose unmanned Exit A. The consistency of exit selection resulted in no significant difference in the influence of gender factors (*p* > 0.05); In scenario 3, *F* = 3.742 and *p* = 0.055, the influence of gender on exit choice is still not significant. The results indicate that when the crowd in the scene is in a non-crowded state, the occurrence of gender effects will significantly affect whether male and female participants follow the crowd’s exit decision. But when there are crowded crowds in the scene, the impact of gender on the herd effect is significantly reduced.

**Table 6:** One-way variance test on exit selection.

No.	Crowd flows	Gender	Selection of exit A	Selection of exit B	*F*	*P*
1	0 vs. 10	Male	51 (78.46%)	14 (21.54%)	6.799	0.01*
		Female	38 (57.58%)	28 (42.42%)		
2	0 vs. 25	Male	64 (98.46%)	1 (1.54%)	1.822	0.179
		Female	63 (93.94%)	4 (6.06%)		
3	10 vs. 25	Male	64 (98.46%)	1 (1.54%)	3.742	0.055
		Female	60 (90.91%)	6 (9.09%)		

No. 1 represent the scenario 1; No. 2, 3 represent the scenario 2, 3.

Then, Table 7 explored the mutual influence between gender factors and different context effects. We found that under the influence of similarity and attraction effects, the exit selection of the male population showed significant changes in statistical significance (Sig _similarity A1_ = 0.001, Sig _similarity A2_ = 0.002, Sig _similarity A3_ = 0.000, Sig _similarity B1_ = 0.000, Sig _attraction A2_ = 0.049, Sig _attraction A3_ = 0.004, Sig _attraction B3_ = 0.033). When the female population was only affected by similarity effects, exit selection was significantly affected (Sig _similarity A2_ = 0.003, Sig _similarity A3_ = 0.026, Sig _similarity B1_ = 0.018). In some scenarios, similarity effects and attraction effects may not have a significant impact on export selection, which may be due to the consistent effect of situational effects on the original export decision (Sig _similarity B2_ = 1.000, Sig _similarity B3_ = 0.321, Sig _attraction A1_ = 0.088, Sig _attraction B1_ = 0.370, Sig _attraction B1_ = 0.418). There is a significant difference in the mutual influence between gender effects and context effects on people’s exit decisions, with males being significantly more affected by context effects than females.

**Table 7:** The statistics of gender effect under different context.

Gender	The context effect	Scenarios	σ	S.E.	*t*	Sig
Male	The similarity effect of exit A	Scenario 1	−0.36923	0.83981	−3.545	0.001*
		Scenario 2	−0.29231	0.72291	−3.260	0.002*
		Scenario 3	−0.46154	0.84921	−4.382	0.000*
	The similarity effect of exit B	Scenario 1	−0.30769	0.63549	−3.904	0.000*
		Scenario 2	0.00000	0.17678	0.000	1.000
		Scenario 3	−0.03077	0.24807	−1.000	0.321
	The attraction effect of exit A	Scenario 1	−0.12308	0.57303	−1.732	0.088
		Scenario 2	−0.13846	0.55557	−2.009	0.049*
		Scenario 3	−0.23077	0.63169	−2.945	0.004*
	The attraction effect of exit B	Scenario 1	−0.04615	0.41196	−0.903	0.370
		Scenario 2	−0.03077	0.30461	−0.814	0.418
		Scenario 3	−0.09231	0.34110	−2.182	0.033*
Female	The attraction effect of exit A	Scenario 1	−0.18182	0.94314	−1.566	0.122
		Scenario 2	−0.25758	0.68636	−3.049	0.003*
		Scenario 3	−0.21212	0.75478	−2.283	0.026*
	The attraction effect of exit B	Scenario 1	−0.21212	0.71285	−2.417	0.018*
		Scenario 2	−0.09091	0.45496	−1.623	0.109
		Scenario 3	−0.04545	0.44486	−0.830	0.410
	The attraction effect of exit A	Scenario 1	0.09091	0.62579	1.180	0.242
		Scenario 2	0.00000	0.35082	0.000	1.000
		Scenario 3	−0.03030	0.49520	−0.497	0.621
	The attraction effect of exit B	Scenario 1	0.00000	0.58177	0.000	1.000
		Scenario 2	−0.01515	0.32781	−0.375	0.709
		Scenario 3	0.00000	0.39233	0.000	1.000

*Sig < 0.05.

Finally, Table 8 specifically analyzed the impact of gender effects on participant evacuation efficiency, and the data results showed that gender effects have a significant impact on evacuation efficiency (*p* = 0.000). Through one-way analysis of variance, it was found that the overall evacuation time (*F* = 132.987, *p* = 0.000), reaction time (*F* = 34.433, *p* = 0.000), and evacuation speed (*F* = 461.611, *p* = 0.000) of participants of different genders all had a significant impact on statistical significance.

**Table 8:** One-way variance test of the effect of sex effect on evacuation efficiency.

Variables	Gender	Mean value	*F*	*P*
Evacuation time	Male	26.13	132.987	0.000*
	Female	31.30		
Reaction time	Male	1.26	34.433	0.000*
	Female	1.12		
Speed	Male	1.17	461.611	0.000*
	Female	0.95		

*p-value < 0.05.

### 3.4 The context effect

#### 3.4.1 The similarity effect

When there is an exit C_A_ similar to the original Exit A in the scene, the difference detection statistical results of the similarity effect in different scenarios are shown in Table 9. Through *t*-test, it can be found that in scenario 1, *t* = −3.515 and Sig = 0.001, in scenario 2, *t* = −4.479 and Sig = 0.000, and in scenario 1, *t* = −4.478 and Sig = 0.000, similar effects have a significant impact on the exit choices of participants in all three scenarios (*p* < 0.01). This indicates that similarity effects have a significant impact on crowd exit choices in emergency evacuation, leading to different exit decision-making preferences among participants.

**Table 9:** A *t*-test for the effect of similarity effect A on exit selection.

Scenarios	σ	S.E.	95%CI		*t*	Sig
			Lower	Upper		
Scenario 1	0.895	0.078	−0.429	0.120	−3.515	0.001*
Scenario 2	0.702	0.061	−0.396	0.153	−4.479	0.000*
Scenario 3	0.810	0.071	−0.476	0.196	−4.478	0.000*

Scenario 1 represents there is no people near the Exit A, 10 people near the Exit B. Scenario 2 represents there is no people near the Exit A, 25 people near the Exit B. Scenario 3 represents there is 10 people near the Exit A, 25 people near the Exit B.

*Sig < 0.05.

**Figure 11:**
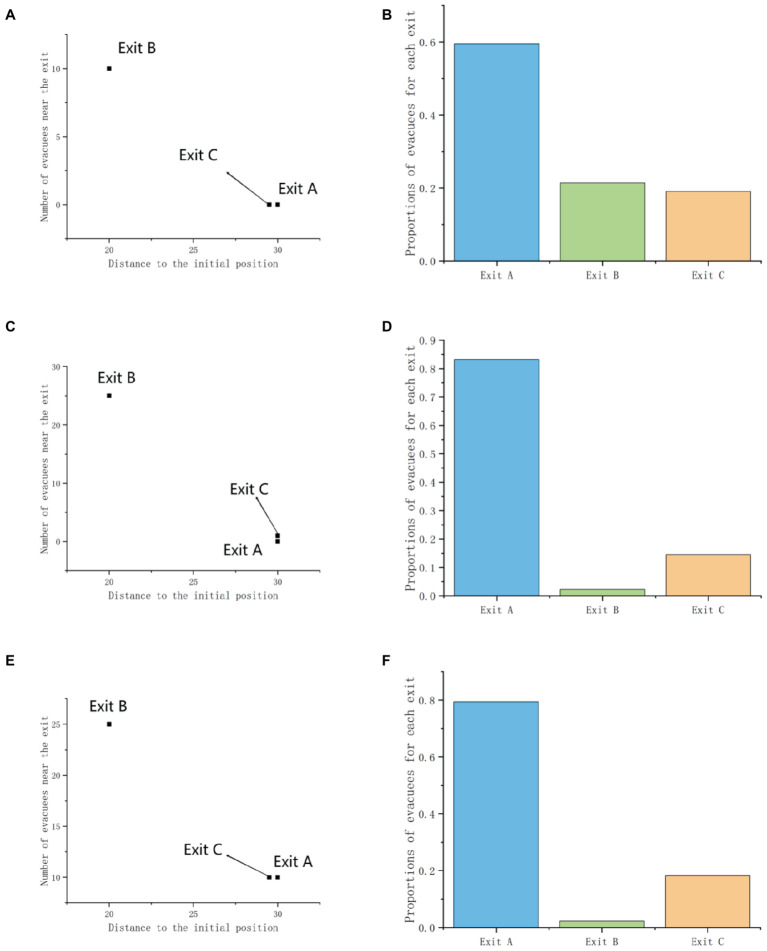
Demonstration of similarity effects: **(A,C,E)** describe the scenario, and **(B,D,F)** show the proportions used at each outlet.

Table 10 further compares and analyzes the exit decisions of different gender groups, and finds that the similarity effect has a significant impact on the exit decisions of both male and female groups in Scenario 2 and Scenario 3 (Sig_2 male_ = 0.002, Sig_2 female_ = 0.003; Sig_3 male_ = 0.000, Sig_3 female_ = 0.026), and the impact on male sex is greater than that on female sex. In scenario 1, the similarity effect only had a significant impact on the male population (Sig = 0.001), and had no significant impact on the female population (Sig = 0.122 > 0.05). This may be due to the fact that when the effect of similar Exit C_A_ is generated, it drives more participants to choose the original Exit B. In Experiment 1, it was found that Scenario 2 and Scenario 3 have fewer choices for Exit B and women are more willing to follow the crowd to choose Exit B. Therefore, similar effects are more significant in Scenario 2, Scenario 3, and men in this experiment (Figure 11).

**Table 10:** Analysis of the interaction between similarity effect A and Gender effects.

	Gender	Exit A	Exit B	Exit C	*t*	Sig.
1	Male	39 (60%)	14 (21.54%)	12 (18.46%)	−3.545	0.001*
	Female	39 (59.09%)	14 (21.21%)	13 (19.70%)	−1.566	0.122
2	Male	54 (83.08%)	2 (3.08%)	9 (13.85%)	−3.260	0.002*
	Female	55 (83.33%)	1 (1.52%)	10 (11.15%)	−3.049	0.003*
3	Male	49 (75.38%)	1 (1.54%)	15 (23.08%)	−4.382	0.000*
	Female	55 (83.33%)	2 (3.03%)	9 (13.64%)	−2.283	0.026*

No. 1 represent the scenario 1; No. 2, 3 represent the scenario 2, 3.

*Sig < 0.05.

When the similarity effect is manifested as the presence of Exit C_B_ similar to the original Exit B in the scene, the difference detection statistical results of the similarity effect in different scenarios are shown in Table 11. Through *t*-test, it can be found that in scenario 1, *t* = −4.403 and Sig = 0.000, in scenario 2, *t* = −1.507 and Sig = 0.134, and in scenario 1, *t* = −1.215 and Sig = 0.227. When similar exits were changed to C_B_, the similarity effect only had a significant impact on the participants’ exit choices in scenario 1 (*p* = 0.000). This is because C_B_ is similar to the original Exit B, and the similarity effect guides the population to choose exit A more and reject exit B. In Experiment 1, the presence of overcrowding in Scenario 2 and Scenario 3 resulted in over 90% of the population choosing Exit A, which is consistent with the decision-making preferences brought about by similar effects, and significant differences cannot occur.

**Table 11:** A *t*-test for the effect of similarity effect B on exit selection.

Scenarios	σ	S.E.	95%CI		*t*	Sig
			Lower	Upper		
Scenario 1	0.675	0.059	−0.376	0.143	−4.403	0.000*
Scenario 2	0.348	0.030	−0.106	0.014	−1.507	0.134
Scenario 3	0.360	0.031	−0.100	0.024	−1.215	0.227

Scenario 1 represents there is no people near the Exit A, 10 people near the Exit B. Scenario 2 represents there is no people near the Exit A, 25 people near the Exit B. Scenario 3 represents there is 10 people near the Exit A, 25 people near the Exit B.

*Sig < 0.05.

**Figure 12:**
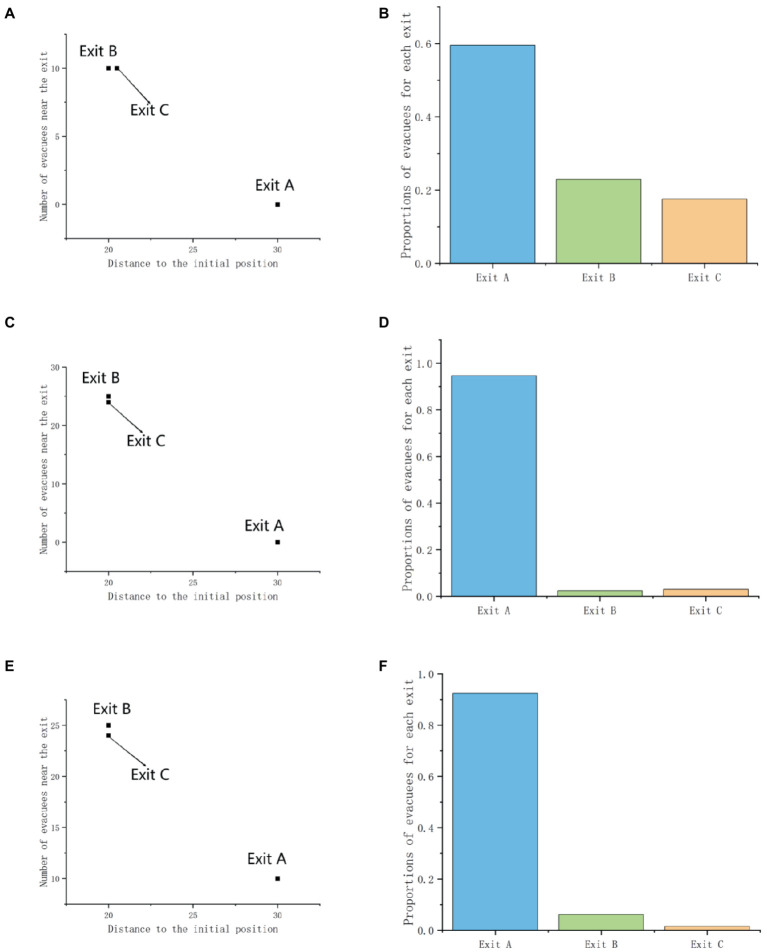
Demonstration of similarity effects: **(A,C,E)** describe the scenario, and **(B,D,F)** show the proportions used at each outlet.

Further analysis of the interaction between gender effects and similarity effects in Table 12 reveals that in Scenario 1, Sig _males_ = 0.000 and Sig _females_ = 0.018, there is no significant difference in exit choices between men and women in both Scenario 2 and Scenario 3 (Sig _males2_ = 1.000 and Sig _females2_ = 0.109; Sig _males3_ = 0.321 and Sig _females3_ = 0.410), with similarity effects having a greater impact on males than females. Females still choose Exit B and Exit C_B_ more than males. This reflects that under the simultaneous influence of herd effect and similarity effect, women are still more willing to follow the crowd to make exit decisions, while men are more affected by environmental factors (Figure 12).

**Table 12:** Analysis of the interaction between similarity effect B and Gender effects.

	Gender	Exit A	Exit B	Exit C	*t*	Sig.
1	Male	41 (63.08%)	14 (21.54%)	10 (15.38%)	−3.904	0.000*
	Female	37 (56.06%)	16 (24.24%)	13 (19.70%)	−2.417	0.018*
2	Male	64 (98.46%)	1 (1.54%)	0 (0.00%)	0.000	1.000
	Female	60 (90.91%)	2 (3.03%)	4 (6.06%)	−1.623	0.109
3	Male	63 (96.92%)	1 (1.54%)	1 (1.54%)	−1.000	0.321
	Female	58 (87.88%)	7 (10.61%)	1 (1.52%)	−0.830	0.410

No. 1 represent the scenario 1; No. 2, 3 represent the scenario 2, 3.

*Sig < 0.05.

#### 3.4.2 The attraction effect

When there is an Exit C_A_ inferior to the original Exit A in the scene, the difference in exit selection caused by the attraction effect is shown in Table 13. In Scenario 1 and Scenario 2, the attraction effect did not show significant differences in the exit choices of participants (Sig1 = 0.774, Sig2 = 0.095). In scenario 3, the attraction effect had a significant impact on the exit choices of participants (Sig = 0.011). This may be because the population is influenced by context factors, and the existence of Exit C_A_ actually diverts exit choices, resulting in a decrease in the choice of Exit A in scenario 3, which is opposite to the exit preference that the attraction effect aims to achieve.

**Table 13:** A *t*-test for the effect of attraction effect A on exit selection.

Scenarios	σ	S.E.	95%CI		*t*	Sig
			Lower	Upper		
Scenario 1	0.607	0.053	−0.120	0.090	−0.288	0.774
Scenario 2	0.467	0.041	−0.149	0.012	−1.683	0.095
Scenario 3	0.574	0.050	−0.229	0.031	−2.589	0.011*

Scenario 1 represents there is no people near the Exit A, 10 people near the Exit B. Scenario 2 represents there is no people near the Exit A, 25 people near the Exit B. Scenario 3 represents there is 10 people near the Exit A, 25 people near the Exit B.

*Sig < 0.05.

**Figure 13:**
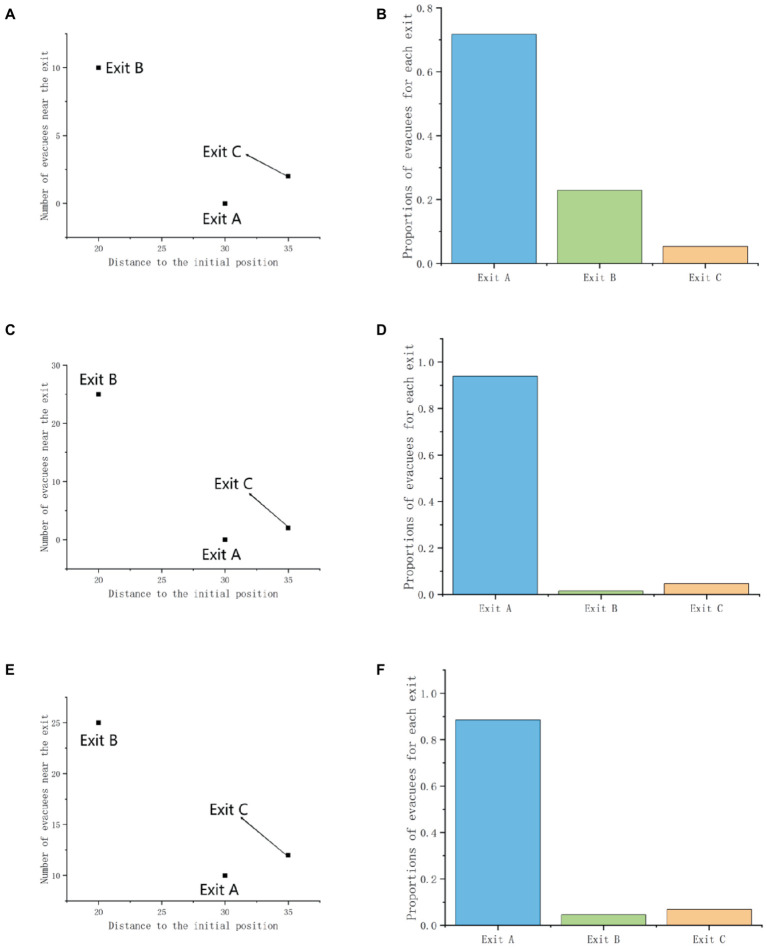
Demonstration of attraction effects: **(A,C,E)** describe the scenario, and **(B,D,F)** show the proportions used at each outlet.

Further analysis of the exit decision preferences brought about by the interaction between gender effects and context effects in Table 14 reveals that (Sig_1 male_ = 0.088, Sig_1 female_ = 0.242; Sig_2 male_ = 0.049, Sig_2 female_ = 1.00; Sig_3 male_ = 0.004, Sig_3 female_ = 0.621), the attraction effect has a significantly greater impact on males than females. The data from Experiment 1 reflects that when there are crowded crowds in the scene, more people choose Exit A that is far away but not crowded. However, in this experiment, it was found that men’s exit choices showed significant changes in both situations (Sig_2 male_ = 0.049, Sig_3 male_ = 0.004). Due to the lower degree of congestion at Exit C_A_ compared to Exit B, even slightly further away from Exit A, male exit choices will be significantly diverted by Exit C. This reflects that men are less sensitive to distance during emergency evacuation and tend to prefer independent evacuation (Figure 13).

**Table 14:** Analysis of the interaction between attraction effect A and Gender effects.

No	Gender	Exit A	Exit B	Exit C	*t*	Sig.
1	Male	47 (72.31%)	14 (21.54%)	4 (6.15%)	−1.732	0.088
	Female	47 (71.21%)	16 (24.24%)	3 (4.55%)	1.180	0.242
2	Male	60 (91.31%)	0 (0.00%)	5 (7.69%)	−2.009	0.049*
	Female	63 (95.45%)	2 (3.03%)	1 (1.52%)	0.000	1.000
3	Male	56 (86.15%)	2 (3.08%)	7 (10.77%)	−2.945	0.004*
	Female	60 (90.91%)	4 (6.06%)	2 (3.03%)	−0.497	0.621

No. 1 represent the scenario 1; No. 2, 3 represent the scenario 2, 3.

*Sig < 0.05.

When there is an Exit C_B_ inferior to the original Exit B in the scene, the difference in exit selection caused by the attraction effect is shown in Table 15. We can observe that the attraction effect (t1 = −0.521, Sig1 = 0.603; t2 = −0.831, Sig2 = 0.407; t3 = −1.420, Sig3 = 0.158) is not significant in all three scenarios. When C_B_ exists, the attraction effect should guide more people to change their exit preferences and choose the original Exit B, but the change in the exit decision is not significant. This reflects that when both attraction effect and herd effect affect people’s exit choice preferences, the impact of attraction effect is lower than that of herd effect.

**Table 15:** A *t*-test for the effect of attraction effect B on exit selection.

Scenarios	σ	S.E.	95%CI		*t*	Sig
			Lower	Upper		
Scenario 1	0.503	0.044	−0.110	0.064	−0.521	0.603
Scenario 2	0.315	0.028	−0.077	0.032	−0.831	0.407
Scenario 3	0.369	0.032	−0.110	0.018	−1.420	0.158

Scenario 1 represents there is no people near the Exit A, 10 people near the Exit B. Scenario 2 represents there is no people near the Exit A, 25 people near the Exit B. Scenario 3 represents there is 10 people near the Exit A, 25 people near the Exit B.

**Figure 14:**
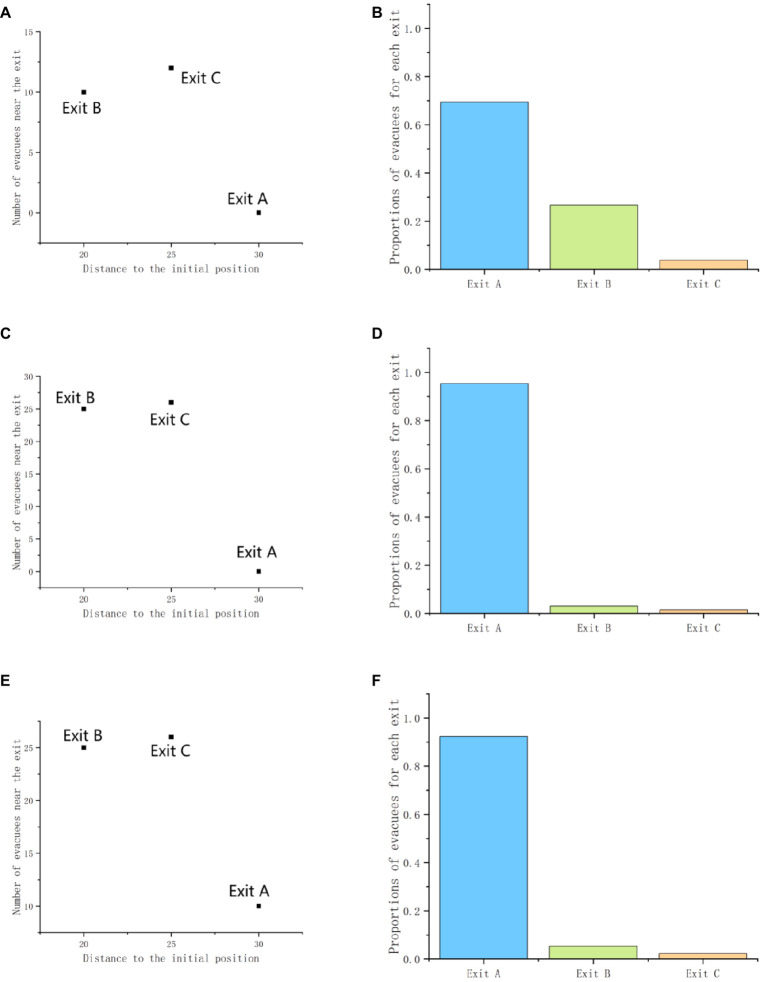
Demonstration of attraction effects: **(A,C,E)** describe the scenario, and **(B,D,F)** show the proportions used at each outlet.

Further analysis of the interaction between gender effect and attraction effect in Table 16 reveals that (Sig_1 male_ = 0.370, Sig_1 female_ = 1.00; Sig_2 male_ = 0.418, Sig_2 female_ = 0.709; Sig_3 male_ = 0.033, Sig_3 female_ = 1.00) attraction effect has a greater impact on males than females. In scenario 3 (Sig_3 male_ = 0.033 > 0.05), there was a significant change in male exit decision-making. Compared to Experiment 1, males had an increase in their choice of Exit B, which was consistent with the exit preference brought about by the attraction effect. In this experiment, women’s choice of Exit B actually decreased, which contradicts the exit preference brought about by similar effects (Figure 14).

**Table 16:** Analysis of the interaction between attraction effect A and Gender effects.

No	Gender	Exit A	Exit B	Exit C	*t*	Sig.
1	Male	49 (75.38%)	15 (23.08%)	1 (1.54%)	−0.903	0.370
	Female	42 (63.64%)	20 (30.30%)	6 (6.06%)	0.000	1.000
2	Male	63 (96.92%)	1 (1.54%)	1 (1.54%)	−0.814	0.418
	Female	62 (93.94%)	3 (4.55%)	1 (1.52%)	−0.375	0.709
3	Male	59 (90.77%)	5 (7.69%)	1 (1.54%)	−2.182	0.033*
	Female	62 (93.94%)	2 (3.03%)	2 (3.03%)	0.000	1.000

No. 1 represent the scenario 1; No. 2, 3 represent the scenario 2, 3.

*Sig < 0.05.

## 4 Discussion

This study explores the effects of crowd flows, gender, and context factors on pedestrian exit selection. It was found that all three factors affect people’s exit choices. Specifically, we found that in emergency evacuation, exit decisions for female groups are more influenced by crowd effects, while context effects are more pronounced for male groups. For example, in the attraction effect in Experiment 2, there was a significant change in male exit choice in both situations (Sig2 male = 0.049, Sig3 male = 0.004), while female exit choice did not show a significant change (Sig > 0.05). Overall, this study found the performance of gender effects in herd and context effects. Previous studies have found that gender has a statistically significant impact on participants’ exit choices (Chaplin, 2015). Female participants are more likely than male participants to follow the majority of participants (Feng et al., 2021), while male participants are better at observing the surrounding environment and conducting independent evacuation (Feng et al., 2020). However, this gender effect will be treated with caution. This article delves into gender specific factors and finds that when pedestrians choose exits, regardless of environmental conditions, the presence of context effects has a significantly higher impact on males than females. This means that during emergency evacuation, women have a stronger willingness to follow the crowd in making exit choices, while men are more inclined to make exit decisions based on environmental factors.

Firstly, women feel more panicked than men, so they are willing to make safer decisions to protect their own safety. According to the data results of Experiment 1, it can be found that there is a significant difference in exit decision-making between women and men in the absence of crowded crowds in the scene (*F* = 6.799, *p* = 0.01), and they are more willing to follow the crowd for evacuation to avoid the uncertainty risk brought by an unselected exit. When the crowd is crowded in the scene, the impact of the herd effect on both women and men is significantly reduced. Both male and female participants tend to choose the exit without crowded crowds (*F* = 1.822, *p* = 0.179; *F* = 3.742, *p* = 0.055), even if no one chooses the exit. This conclusion can be found that women have stronger social attributes than men, therefore women are more willing to make exit decisions through social demographic factors. In contrast, male participants showed less exit choice following the crowd in the experiment and were more influenced by environmental effects. This research result is consistent with previous studies showing that men are more calm during evacuation while women are more likely to feel panic (Lu et al., 2011; Pan et al., 2021).

In addition, gender effects have a significant impact on evacuation efficiency (*p* = 0.000). Previous studies have found that gender has a significant impact on evacuation efficiency (Huang et al., 2022), with males having longer reaction times than females but also accompanied by faster evacuation speeds at the beginning of evacuation. This gender effect is reflected in the emergency evacuation in this article. The average reaction time of women (*M* = 1.12 s) is lower than that of men (*M* = 1.26 s). This is because men tend to observe the environment and make rational exit decisions, while women are more influenced by crowd factors. In terms of overall evacuation time, women (*M* = 31.30 s) were significantly higher than men (*M* = 26.13 s), so the average evacuation speed of women (*M* = 0.95 m/s) was significantly lower than that of men (*M* = 1.17 m/s). This reflects that men have higher evacuation efficiency than women in emergency evacuation and have higher distance accessibility within the space. Table 17 shows that gender has a significant impact on evacuation time (Sig = 0.047), reaction time (Sig = 0.003), and evacuation speed (Sig = 0.000), and different crowd flows in different scenarios also have a significant impact on reaction time (Sig = 0.023) and evacuation speed (Sig = 0.008). This confirms that gender effects and crowd flows have significant statistical benefits on people’s evacuation efficiency, while context effects have a greater impact on people’s exit choices, so they are not significant in terms of evacuation efficiency (Sig > 0.05).

**Table 17:** Multivariate logistic regression analysis on evacuation efficiency.

Predictor	χ^2^	df	Sig
Evacuation time			
Intercept	0.000	0	NA
Gender	41.589	28	0.047*
Scenarios difference-Crowd flow	65.917	56	0.171
The context effect	93.794	112	0.893
Goodness-of fit test	0.000	616	1.000
Reaction time			
Intercept	0.000	0	NA
Gender	38.753	18	0.003*
Scenarios difference-Crowd flow	54.763	36	0.023*
The context effect	85.412	72	0.134
Goodness-of fit test	4.036	396	1.000
Speed			
Intercept	0.000	0	NA
Gender	44.361	14	0.000*
Scenarios difference-Crowd flow	49.041	28	0.008*
The context effect	70.464	56	0.092
Goodness-of fit test	0.000	266	1.000

Secondly, according to the study of context effects in Experiment 2, it can be found that context effects have a statistically significant impact on the exit choice of the population (Gao et al., 2023). However, in this article, there are differences in the impact and degree of influence of these two effects on participant decision-making. In emergency evacuation situations, the similarity effect has a more significant impact on pedestrian exit selection than the attraction effect. Meanwhile, the impact of context effects is more significant in the exit decisions of male participants. The two corresponding intensities of action were not explained in previous studies. This article found through experiments that different effects in situational effects have different impacts on the population, and even have differences in gender. When there is an Exit C_A_ similar to Exit A in the scene, participants will increase their choice of the original Exit B due to the influence of similarity effects. In all three scenarios, the exit choices of participants showed significant changes compared to the situation without similar effects in Experiment 1 (Sig1 = 0.001; Sig2 = 0.000; Sig3 = 0.000), and more participants were influenced by similar Exit *C_A_.* The population who originally chose Exit A was successfully diverted to C_A_ and Exit B. When the similarity effect is manifested as the addition of Exit C_B_ similar to the original Exit B in the scene, it only has a significant impact on the participants’ exit choices in scenario 1 (Sig1 = 0.000; Sig2 = 0.134; Sig3 = 0.227). This is because the effect brought by Exit C_B_ will guide the population to choose Exit A more and reject Exit B. In Experiment 1, there is a crowded population in Scenario 2 and Scenario 3, which leads to over 90% of the population choosing Exit A. This is consistent with the decision-making preferences brought about by similar effects, and significant differences cannot occur. The fact that more participants choose Exit A also reflects the effectiveness of similar effects.

**Figure 15:**
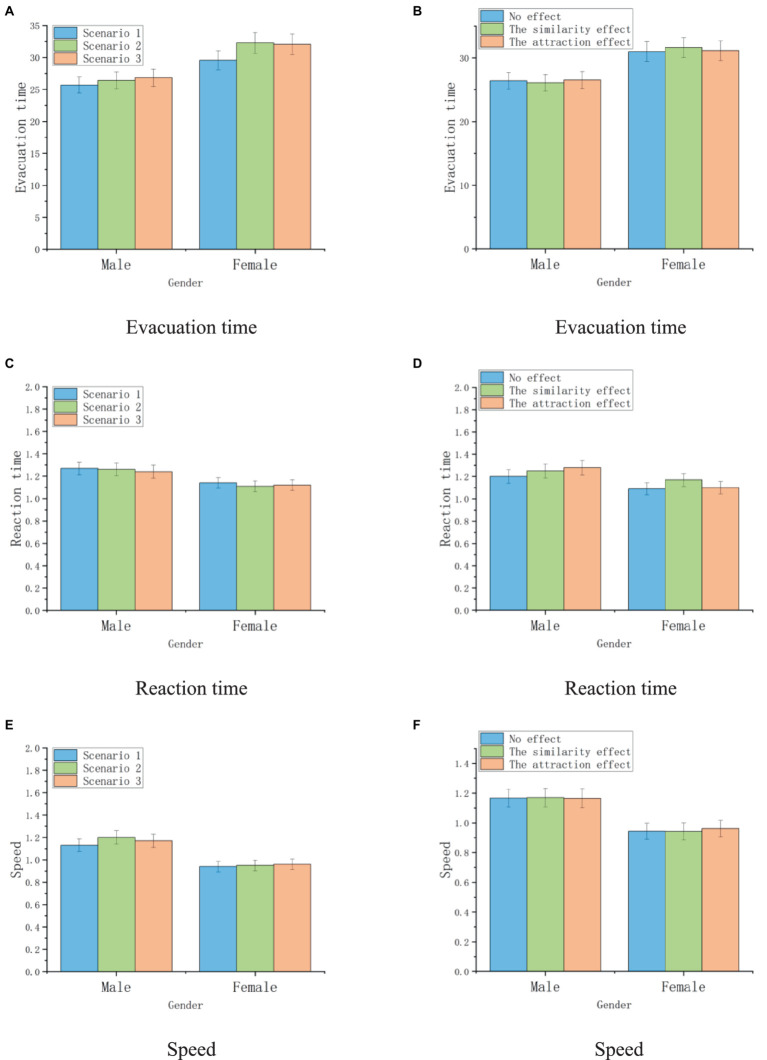
The statistics of participants evacuation efficiency: **(A,C,E)** show the evacuation efficiency under different crowd flow. **(B,D,F)** show the evacuation efficiency under different context effect.

Further analysis of the interaction between similarity effects and gender factors on exit choice reveals that similarity effects have a higher impact on male participants than female participants. At present, there is no article elaborating on the final results of the interaction between context effects and gender effects. In Experiment 2, the following results were obtained by incorporating considerations of context and gender factors. The existence of similar Exit C_A_ has a significant impact on the exit decisions of both male and female groups in Scenario 2 and Scenario 3 (Sig2 male = 0.002, Sig2 female = 0.003; Sig3 male = 0.000, Sig3 female = 0.026), and it can be found that the impact on males is greater than that on females. This may be due to the similarity effect caused by similar Exit C_A_, which affects more participants to choose Exit B. In Experiment 1, it was found that Scenario 2 and Scenario 3 had very few choices for Exit B. Therefore, in this experiment, the similarity effect was more significant in Scenario 2 and Scenario 3. In scenario 1, the similarity effect only had a significant impact on the male population (Sig = 0.001), and had no significant impact on the female population (Sig = 0.122 > 0.05). The different results brought about by this gender difference reflect that the similar effect has a more significant impact on male participants compared to female participants. This research result also confirms that in emergency evacuation environments, men have a stronger ability to receive environmental information than women (Shiwakoti et al., 2016; Figure 15).

Finally, through studying the attraction effect in emergency evacuation situations, it was found that the attraction effect has a less significant impact on the exit choices of participants. When there are inferior Exit C_A_ similar to the original Exit A in the scene, the exit preference brought by the attraction effect should guide more participants to choose original Exit A. However, in Scenario 1 and Scenario 2, the attraction effect did not show a significant difference in the exit choices of participants (Sig1 = 0.774, Sig2 = 0.095). This has not been reflected in previous research (Tong and Bode, 2023), and in this article, the influence of attraction effect is significantly weaker than that of similarity effect. This may be due to the fact that a large number of participants choose Exit A when there is no attraction effect, while the attraction effect is consistent with the original participants’ exit choices. Therefore, significant differences in exit choices cannot be generated, which also reflects the correct reflection of similar effects in this scenario. In scenario 3, the attraction effect had a significant impact on the participants’ exit choices (Sig = 0.011), but the proportion of choices for Exit A actually decreased. Further analysis of the same outcome may be due to the influence of context factors on the population, and the existence of Exit C_A_ actually diverts exit choices, resulting in a decrease in the choice of Exit A in scenario 3, which is opposite to the exit preference that the attraction effect aims to achieve. When there is a lower quality Exit C_B_ similar to the original Exit B in the scene, we can find that the attraction effect (Sig1 = 0.603; Sig2 = 0.407; Sig3 = 0.158) is not significant in all three situations. When C_B_ exists, the attraction effect should guide more people to change their exit preferences and choose the original B exit, but the change in the exit decision is not significant. This reflects that the impact of attraction effect on emergency evacuation is not significant, and more participants will still choose to avoid crowded Exits B and C_B_. In this situation, the impact of attraction effect is lower than that of herd effect.

Furthermore, similar to the similarity effect, the attraction effect also has a more significant impact on male participants. When there is an attractive Exit C_A_ (Sig_1 male_ = 0.088, Sig_1 female_ = 0.242; Sig_2 male_ = 0.049, Sig_2 female_ = 1.00; Sig_3 male_ = 0.004, Sig_3 female_ = 0.621), the impact of attraction on males is significantly greater than that on females. There were significant changes in the exit choices of males in both Scenario 2 and Scenario 3 (Sig_2 males_ = 0.049, Sig_3 males_ = 0.004). Due to the lower degree of congestion in Exit C_A_ compared to Exit B, even slightly further away from Exit A, male exit choices will be significantly diverted by Exit *C_A_.* This indicates that men are more inclined to avoid crowd evacuation during emergency evacuation, even if the exit is far away, reflecting that male participants have lower sensitivity to exit distance and are more inclined to evacuate independently in emergency evacuation situations. This difference in gender perception of distance has also been reflected in previous research(Lu et al., 2011). When adding attractive exit C_B_ (Sig_1 male_ = 0.370, Sig_1 female_ = 1.00; Sig_2 male_ = 0.418, Sig_2 female_ = 0.709; Sig_3 male_ = 0.033, Sig_3 female_ = 1.00), the impact of attraction on males is also greater than that on females. In scenario 3 (Sig_3 male_ = 0.033 > 0.05), there was a significant change in male exit decision-making, and the attraction effect increased the choice of B exit. In this experiment, women’s choice of Exit B actually decreased, which contradicts the exit preference brought about by similar effects.

There are some limitations in this article that need to be pointed out and addressed in future research. Firstly, we investigated pedestrian exit selection in a virtual environment. Although some previous work has directly demonstrated the effectiveness of virtual experimental research on pedestrian exit selection and decision-making in certain situations, this experiment does not study the decision-making of participants in the real world. In addition, the explanations provided in the laboratory, the steering mechanisms of the mouse and keyboard, and environmental characteristics are all aspects that may affect the decision-making of participants. Because they are fixed in the experiment, they do not affect the internal validity of our experiment. However, further real-world data investigation is needed to determine whether our findings can be extended to real-world human exit choices. Secondly, the impact of context effects in this article is only a basic study and cannot reveal in depth the preference changes or other behavioral changes brought about by context effects in more complex environments. Therefore, the role of context can be further explored in future research. Finally, this study mainly explored the influence of gender on exit choice, but there may also be potential influences from factors such as the personality and personal experience of the participants. This potential impact will be further explored in future research.

## 5 Conclusion

This study confirms the importance of gender factors in influencing exit choice behavior through their interaction with context and herd effects. The research results show that overall, the female population is more affected by the herd effect in exit choices, and is more willing to follow the population for exit choices than males; The male population is more affected by context effects and tends to engage in independent evacuation. Specifically, the crowd naturally has a preference for avoiding crowded crowds. When there is a crowded crowd near the exit, participants are more willing to evacuate by avoiding the crowd, even if there are no other exits to choose from. The context effect, as an environmental factor, has a more significant impact on the male population, while the similarity effect has a greater impact on the participants’ exit choices than the attraction effect. The existence of similar exits can effectively change the exit decisions of participants, which also provides reference significance for the exit design of architectural scenes.

## Data availability statement

The original contributions presented in the study are included in the article/supplementary material, further inquiries can be directed to the corresponding author/s.

## Ethics statement

The studies involving humans were approved by The Ethics Committee of Sichuan Normal University approved the research plan in accordance with the Helsinki International Declaration. The studies were conducted in accordance with the local legislation and institutional requirements. The participants provided their written informed consent to participate in this study.

## Author contributions

QT: Writing – original draft, Methodology, Investigation, Conceptualization. XW: Writing – review & editing, Conceptualization. WH: Writing – review & editing, Data curation, Conceptualization. GP: Writing – review & editing, Project administration, Formal analysis. YM: Writing – review & editing, Supervision, Funding acquisition, Conceptualization.
